# Taba Binary, Multinomial, and Ordinal Regression Models: New Machine Learning Methods for Classification

**DOI:** 10.3390/bioengineering12010002

**Published:** 2024-12-24

**Authors:** Mohammad Tabatabai, Derek Wilus, Chau-Kuang Chen, Karan P. Singh, Tim L. Wallace

**Affiliations:** 1 School of Global Health, Meharry Medical College, Nashville, TN 37208, USA; 2 School of Medicine, University of Texas at Tyler, Tyler, TX 75708, USA; karan.singh@uttyler.edu; 3 School of Applied Computational Sciences, Meharry Medical College, Nashville, TN 37208, USA; twallace@mmc.edu

**Keywords:** artificial neural network, random forest, logistic regression, probit analysis, machine learning, classification

## Abstract

The classification methods of machine learning have been widely used in almost every discipline. A new classification method, called Taba regression, was introduced for analyzing binary, multinomial, and ordinal outcomes. To evaluate the performance of Taba regression, liver cirrhosis data obtained from a Mayo Clinic study were analyzed. The results were then compared with an artificial neural network (ANN), random forest (RF), logistic regression (LR), and probit analysis (PA). The results using cirrhosis data revealed that the Taba regression model could be a competitor to other classification models based on the true positive rate, F-score, accuracy, and area under the receiver operating characteristic curve (AUC). Taba regression can be used by researchers and practitioners as an alternative method of classification in machine learning. In conclusion, the Taba regression provided a reliable result with respect to accuracy, recall, F-score, and AUC when applied to the cirrhosis data.

## 1. Introduction

Classification is a supervised technique of categorizing a given set of data into classes using one or more variables. Classification models are predictive methods that assign predefined categories to cases based on the characteristics of these cases. These methods are widely used in all areas of science, medicine, engineering, and biomedical-related or science-related fields. Over the years, classification methods have progressed meaningfully, observing the expansion of Bayesian classification [1], decision trees, and support vector machines [2]. Cluster analysis is another machine learning (ML) method used to identify subgroups in a given dataset, especially datasets with high dimensionality. There are numerous algorithms for clustering that use different similarity measures [3].

Classification techniques, which have the capacity to handle large datasets, play an important role in almost every field. These models find applications in a wide range of fields including spam detection algorithms [4], bank loan decisions with different levels of risk of default, sentiment analysis, fraud detection systems, and medical diagnosis. By accurately categorizing data, these models help businesses make informed decisions, identify patterns, and gain valuable insights for improved decision-making processes. 

An Artificial Neural Network (ANN) is a machine learning method using the concept of a human neuron. It is a computational method that imitates the way biological neurons work [5]. RF is a group of decision trees in which each tree is trained with a specific random noise. The random aspect of RF reduces the risk of overfitting and improves overall model classification. It also ensures high predictive precision and flexibility [6]. 

Logistic regression (LR) and probit analysis (PA) are classification algorithms that can be used to predict categorical outcome variables using a set of independent variables. The LR assumes that the error terms have a logistic probability distribution, but PA assumes that the error term follows a normal probability distribution [7].

A comparative study of random forest, logistic regression, and neural networks used accuracy and computational efficiency as a tool for their performance in classification [8]. In an earthquake-induced landslide study, RF and ANN models were assessed using accuracy, AUC, precision, specificity, and recall ratio [9]. ML metrics were used to evaluate the model performance of RF and LR [10]. A systematic review measured the overall performance of LR and an ANN [11]. 

ML techniques were used to assess and examine subjects with post-traumatic stress disorder (PTSD) and acute stress disorder (ASD) [12]. A comprehensive systematic review on mental health patients was performed using ML techniques for analyzing bipolar disorder patients [13]. He et al. studied the estimation of depression relating to deep neural networks and Ramos-Lima researched the application of ML techniques in assessing subjects with PTSD and ASD [12,14]. Tabatabai et al. used the binary Hyperbolastic regression of type II to study the role of a patient-centered communication scale on the patient satisfaction of healthcare providers in the USA [15]. In addition, the risk assessment of death classification during the COVID 19 alpha, delta, and omicron periods was evaluated using the Hyperbolastic regression of type I [16].

This paper introduces a new classification method called Taba regression that will enable the user to classify binary, multinomial, and ordinal categorical data. The performance of this method, with respect to ML metrics, was evaluated using liver cirrhosis data in comparison with LR, PA, an ANN, and RF.

## 2. Materials and Methods

### 2.1. Taba Probability Distribution

For the random variable *X*, −∞<x<∞. The standard Taba CDF is defined as

$$F_{Taba}\left(x\right)=\frac{1}{1+{\left[Sinh\left(e^{-ArcSinh\left(x\right)}\right)\right]}^{2}},$$

The Taba CDF can be used as an activation function in a neural network. The inverse of the standard Taba distribution function is given by

$${F_{Taba}}^{-1}\left(F\right)=Sinh\left(-ln\left(ArcSinh\sqrt{\frac{1-F}{F}}\right)\right)\;\;\;\;\;\;\;\;\;\;\;\;\;\;0<F<1\;.$$

The probability density function for the Taba distribution is given by

$$f\left(x\right)=\frac{e^{-ArcSinh\left(x\right)}}{{\left(1+e^{-ArcSinh\left(x\right)}\right)}^{2}\sqrt{1+x^{2}}}$$

**Figure 1:**
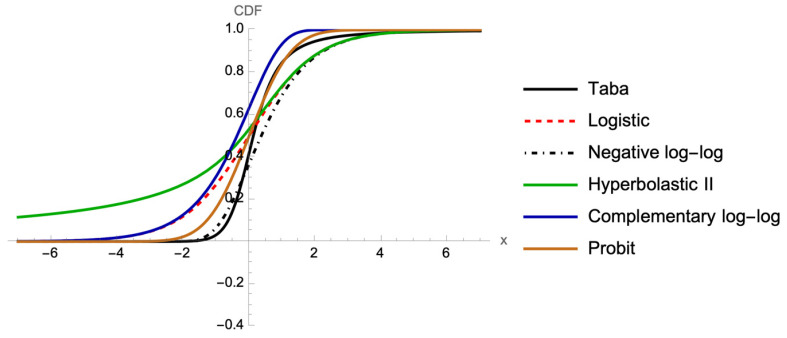
Graph of CDF for $F_{L}$, $F_{p},$ $F_{N}$, $F_{CLL}$, $F_{H_{II}}$, and $F_{Taba}.$

**Figure 2:**
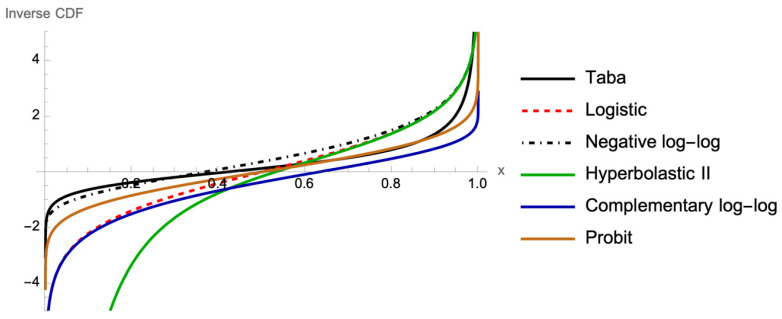
Graph of inverse functions of $F_{L}$, $F_{p},$ $F_{N}$, $F_{CLL}$, $F_{H_{II}}$, and $F_{Taba},.$

Figure 1 shows the CDF of logistic ($F_{L}$), probit ($F_{p}$), negative log-log ($F_{N}$), complementary log-log ($F_{CLL}$), Hyperbolastic II ($F_{H_{II}}$), and Taba ($F_{Taba}$), and Figure 2 illustrates the corresponding graphs of the inverse CDF, or link functions, which are functions of probabilities that result in a linear model in the parameters. 

### 2.2. Taba Regression

The Taba regression is a classification model that utilizes Taba probability distribution to model a binary (dichotomous), multinomial (nominal multiclass), or ordinal (ordinal multiclass) outcome variable. Taba regression is an ML classification method, which is a supervised learning technique. It is used for predicting the categorical dependent variable utilizing a given set of explanatory variables.

#### 2.2.1. Binary Taba Regression

For the binary variable Y, let success be defined as Y=1 and failure as Y=0, then the success probability of a sample of size n as a function of parameter vector $\beta =\left(\beta_{0},\beta_{1},\beta_{2},...,\beta_{p}\right)$ is defined as

(1) $$\pi \left(x_{i};\beta \right)=P\left(y_{i}=1|x_{i}\right)=\frac{1}{1+{\left[Sinh\left(e^{-ArcSinh\left(\beta_{0}{+\beta }_{1}x_{i1}+\beta_{2}x_{i2}+...+\beta_{p}x_{ip}\right)}\right)\right]}^{2}},\;i=1,2,...,n$$

where $x_{i}={\left({1,x}_{i1},x_{i2},...,x_{ip}\right)}^{T}$ and p represents the number of explanatory variables. Now consider the model

$$y_{i}=\pi \left(x_{i};\beta \right)+\epsilon_{i},$$

where $\epsilon_{1},\epsilon_{2},...,\epsilon_{n}$ are independent random variables with expected value $E(\epsilon_{i})=0$ and $y_{1},y_{2},...,y_{n}$ are independent Bernoulli variables with a mean $E\left(y_{i}\right)=\pi \left(x_{i};\beta \right)$ and variance $Var\left(y_{i}\right)=\pi \left(x_{i};\beta \right)\left(1-\pi \left(x_{i};\beta \right)\right)$. 

For simple binary Taba regression with only one explanatory variable, the function (1) reduces to

$$\pi \left(x_{i};\beta \right)=P\left(y_{i}=1|x_{i}\right)=\frac{1}{1+{\left[Sinh\left(e^{-ArcSinh\left(\beta_{0}{+\beta }_{1}x_{i}\right)}\right)\right]}^{2}},$$

or equivalently

$$\pi \left(x_{i};\beta \right)=\frac{1}{1+{\left[Sinh\left(e^{-ArcSinh\left(\frac{x_{i}-L}{S}\right)}\right)\right]}^{2}}$$

where L and S are location and scale parameters with $L=\frac{-\beta_{0}}{\beta_{1}}$ and $S=\frac{1}{\beta_{1}}$.

For binary Taba regression, the odds of success are:

$${Odds}_{Taba}=\frac{1}{{\left[Sinh\left(e^{-ArcSinh\left(\beta_{0}{+\beta }_{1}x_{i1}+\beta_{2}x_{i2}+...+\beta_{p}x_{ip}\right)}\right)\right]}^{2\;}}$$

The purpose of binary Taba regression is to predict a binary outcome by means of a single independent variable (simple binary Taba regression) or a set of independent variables (multiple binary Taba regression). In general, binary classification models are routinely used in many areas including medical, public health, dental, biomedical, machine learning, social, behavioral, and engineering sciences. 

##### Assumptions of Binary Taba Regression

Similar to other binary classification methods, the following assumptions must be satisfied:

- Each observation must be independent of one another.
- The dependent variable must be binary.
- Absence of multicollinearity among independent variables.
- Linear relationship between explanatory variables and hyperbolic sine of log odds.
- There should be no strong outliers, high leverage values, or influential observations in the dataset.

##### Parameter Estimation for Binary Taba Regression

Because Taba regression predicts probabilities, we can estimate weight parameters using likelihood function. For each training datapoint, we have a vector of features, $x_{i}$, and an observed class, $y_{i}$. The probability of that class was either $\pi \left(x_{i};\beta \right)$, if $y_{i}=1$, or $1-\pi \left(x_{i};\beta \right)$, if $y_{i}=0$.

The likelihood function for binary Taba regression is equal to

$$\prod_{i=1}^{n}\left({\left(\pi \left(x_{i};\beta \right)\right)}^{y_{i}}{\left(1-\pi \left(x_{i};\beta \right)\right)}^{1-y_{i}}\right)$$

The estimate of the weight parameter vector $\beta =\left({\beta_{0},\beta }_{1},\beta_{2},...,\beta_{p}\right)$ can be found by maximizing the log-likelihood function of the form

$$\hat{\beta }=\underset{\beta }{\mathrm{argmax}}\sum_{i=1}^{n}\left(y_{i}ln\left(\pi \left(x_{i};\beta \right)\right)+\left(1-y_{i}\right)ln\left(1-\pi \left(x_{i};\beta \right)\right)\right)$$

The estimated variance–covariance matrix for a weight parameter vector is calculated using the inverse of the estimated Fisher’s Information matrix, from which the test statistics are derived.

#### 2.2.2. Multinomial Taba Regression

The multinomial Taba regression is used when the dependent variable is a nominal categorical with more than two levels and there is no natural order. In this model, the explanatory variables can be categorical or continuous.

##### Assumptions of Multinomial Taba Regression

Similar to other multinomial models, multinomial Taba regression must satisfy the following:

- Observations are assumed to be independent.
- Outcome categories are assumed to be mutually exclusive and collectively exhaustive.
- No severe outliers, high leverage values, or highly influential observation in the dataset.
- Lack of multicollinearity between independent variables.
- Linear relationship between independent variables and the hyperbolic *sine* of log odds of success

##### Parameter Estimation for Multinomial Taba Regression

Corresponding with each individual observation i, the outcome variable $y_{i}$ has k categories $y_{i}\in \left\{1,2,...,k\right\}\;$ and $y_{i}$ has a multinomial distribution with probability vector $\left(\pi_{1},\pi_{2},...,\pi_{k}\right)$. The general multinomial model is represented by k−1 indicator variables $y_{ij}$ defined as

$$y_{ij}=\left\{\begin{array}{c} 1\;\;if\;y_{i}=j\\ 0\;\;if\;y_{i}\neq j \end{array}\right.$$

meaning that $y_{ij}=1$ whenever the ith response is in the jth category and 0, otherwise.

Defining the weight parameter vector $\beta_{j}=\left(\beta_{j0},\beta_{j1},...,\beta_{jp}\right)$ and matrix $\beta ={\left(\beta_{1},\beta_{2},...,\beta_{k-1}\right)}^{T}$ with category *r* as a reference category and $\pi_{r}\left(x_{i};\beta \right)$ as its corresponding probability function, the multinomial Taba probabilities are defined for categories j=1,...,k as

$$\pi_{j}\left(x_{i};\beta \right)=\left\{\begin{array}{c} \frac{1}{1+\sum_{\begin{array}{c} s=1\\ s\neq r \end{array}}^{k}{\left[Sinh\left(e^{-ArcSinh\left(\varphi_{s}\left(x_{i};\beta \right)\right)}\right)\right]}^{2}},\;\;\;\;\;\;\;\;\;\;\;\;\;if\;j=r\\ \frac{{\left[Sinh\left(e^{ArcSinh\left(\varphi_{j}\left(x_{i};\beta \right)\right)}\right)\right]}^{2}}{1+\sum_{\begin{array}{c} s=1\\ s\neq r \end{array}}^{k}{\left[Sinh\left(e^{-ArcSinh\left(\varphi_{s}\left(x_{i};\beta \right)\right)}\right)\right]}^{2}},\;\;\;\;\;\;\;\;\;\;\;\;\;if\;j\neq r \end{array}\right.,$$

where $\varphi_{s}\left(x_{i};\beta \right)=\beta_{s0}{+\beta }_{s1}x_{i1}+\beta_{s2}x_{i2}+...+\beta_{sp}x_{ip}$ and i=1,2,...,n.

The weight parameter β then can be estimated using

$$\hat{\beta }=\underset{\beta }{\mathrm{argmax}}\sum_{i=1}^{n}\left[y_{i1}ln\left(\pi_{1}\left(x_{i};\beta \right)\right)+y_{i2}ln\left(\pi_{2}\left(x_{i};\beta \right)\right)+...+y_{ik}ln\left(\pi_{k}\left(x_{i};\beta \right)\right)\right]$$

#### 2.2.3. Ordinal Taba Regression

Ordinal Taba regression, or Taba proportional odds regression, is used to classify an ordinal outcome variable using a set of explanatory variables. Level rankings of the outcome variable categories do not imply equal distances between them.

##### Assumptions of Ordinal Taba Regression

Similar to the proportional odds model assumptions, Taba ordinal regression must satisfy the following:

- The dependent variable must be measured on an ordinal level.
- No independent variable has an unequal effect on a specific level of the ordinal categorical dependent variable.
- The predictor variables must be independent.

To test the validity of the proportional odds model, the score test can be used. The Brant and Wolfe–Gould tests for proportional odds are appropriate when the sample size is small [1,2]. The proportional odds assumption needs to be tested prior to its usage [17,18].

##### Parameter Estimation for Ordinal Taba Regression

For the *k*-category ordinal outcome, the cumulative probability for the *i*th response belongs to the category less than or equal to *j* and is given by

$$F_{j}(x_{i})=P(y_{i}\;\leq j|x_{i}),$$

and for j=1,...,k−1, and $\alpha_{1}<\alpha_{2}<...<\alpha_{k-1}$. The $\gamma_{j}\left(x_{i};\alpha_{j},\beta \right)$ is the ordinal log-odds of falling into or below category j against falling above it and is equal to

$$\gamma_{j}\left(x_{i};\alpha_{j},\beta \right)=ln\left(\frac{F_{j}\left(x_{i}\right)}{1-F_{j}\left(x_{i}\right)}\right)=-2ln\left(Sinh\left(e^{-ArcSinh\left(L_{j}\right)}\right)\right),$$

where ${L_{j}=\alpha }_{j-}\beta_{1}x_{i1}-\beta_{2}x_{i2}...-\beta_{p}x_{ip}$

For parameter vectors $\alpha =\left(\alpha_{1},\alpha_{2},...,\alpha_{k-1}\right)\;and\;\beta =\left(\beta_{1},\beta_{2},...,\beta_{p}\right),$ let ${\pi_{j}}^{O}\left(x_{i};\alpha ,\beta \right)$ denote the probability $P\left(y_{i}=j|x_{i};\alpha ,\beta \right),$ then we have

$${\pi_{j}}^{O}\left(x_{i};\alpha ,\beta \right)=\left\{\begin{array}{c} \frac{1}{1+e^{\gamma_{j}\left(x_{i};\alpha_{j},\beta \right)}},\;\;\;\;\;\;\;\;\;\;\;\;\;\;\;\;\;\;\;\;\;\;\;\;\;\;\;\;\;\;\;if\;j=1\\ \frac{1}{1+e^{\gamma_{j}\left(x_{i};\alpha_{j},\beta \right)}}-\frac{1}{1+e^{\gamma_{j-1}\left(x_{i};\alpha_{j-1},\beta \right)}},\;\;\;\;\;\;\;\;\;\;\;\;\;if\;2\leq j\leq k-1\\ 1-\frac{1}{1+e^{\gamma_{j-1}\left(x_{i};\alpha_{j-1},\beta \right)}},\;\;\;\;\;\;\;\;\;\;\;\;\;\;\;if\;j=k \end{array}\right.,$$

or equivalently

$${\pi_{j}}^{O}\left(x_{i};\alpha ,\beta \right)\left\{\begin{array}{c} \frac{1}{1+{\left[Sinh\left(e^{-ArcSinh\left(L_{j}\right)}\right)\right]}^{2}},\;\;\;\;\;\;\;\;\;\;\;\;\;\;\;\;\;\;\;\;\;\;\;\;\;\;\;\;\;\;\;\;\;\;\;\;\;\;if\;j=1\\ \frac{1}{1+{\left[Sinh\left(e^{-ArcSinh\left(L_{j}\right)}\right)\right]}^{2}}-\frac{1}{1+{\left[Sinh\left(e^{-ArcSinh\left(L_{j-1}\right)}\right)\right]}^{2}},\;\;\;\;\;\;\;if\;2\leq j\leq k-1\\ 1-\frac{1}{1+{\left[Sinh\left(e^{-ArcSinh\left(L_{j-1}\right)}\right)\right]}^{2}},\;\;\;\;\;\;\;\;\;\;\;\;\;\;\;\;\;\;\;\;\;\;\;\;\;\;if\;j=k \end{array}\right..$$

The likelihood function for ordinal Taba regression is

$$\prod_{i=1}^{n}\prod_{j=1}^{k}{\left({\pi_{j}}^{O}\left(x_{i};\alpha ,\beta \right)\right)}^{y_{ij}}$$

and the log-likelihood is

$$\sum_{i=1}^{n}\sum_{j=1}^{k}y_{ij}ln\left({\pi_{j}}^{O}\left(x_{i};\alpha ,\beta \right)\right)$$

The estimate of parameter vectors α,β can be obtained by maximizing the log-likelihood function

$$\hat{\alpha ,\beta }=\underset{\alpha ,\beta }{\mathrm{argmax}}\sum_{i=1}^{n}\left(y_{i1}ln\left({\pi_{1}}^{O}\left(x_{i};\alpha ,\beta \right)\right)+y_{i2}ln\left({\pi_{2}}^{O}\left(x_{i};\alpha ,\beta \right)\right)+...+y_{ik}ln\left({\pi_{jk}}^{O}\left(x_{i};\alpha ,\beta \right)\right)\right)$$

## 3. Results

### 3.1. Analysis of Liver Cirrhosis Data

Cirrhosis results from extended liver damage, causing massive scarring, most likely due to hepatitis or chronic alcohol consumption. The data used here are from a Mayo Clinic study on Primary Biliary Cirrhosis (PBC) of the liver carried out from 1974 to 1984. During this period, 424 PBC patients referred to the Mayo Clinic qualified for the randomized placebo-controlled trial testing the drug D-penicillamine. The Cirrhosis Patient Survival Prediction data were obtained from the UC Irvine repository. Only 312 patients participated in the trial [19,20]. Table 1 shows the demographic and clinical characteristics of the patients. A total of 40.1% died of cirrhosis; 50.6% had received the drug D-Penicillamine and 49.1% were given a placebo; 7.7% had ascites, 51.3% had hepatomegaly, and 28.8% had spiders. 

The mean age for patients was 18,269.44 days, with a standard deviation of 3864.805 days. The age range was from 9598 to 28,650 days. The mean bilirubin level was 3.256 mg/dL, with a standard deviation of 4.530 mg/dL. Albumin had a mean level of 3.52 mg/dL and a standard deviation of 0.41989 mg/dL. The mean cholesterol level was 368.75 mg/dL, with a standard deviation of 233.063 mg/dL, and copper had a mean level of 97.04 μg/dL, with a standard deviation of 86.019 μg/dL. Alkaline phosphatase had a mean level of 1982.66 IU/L and a standard deviation of 2140.389 IU/L, as shown in Table 2.

#### 3.1.1. Analysis of Cirrhosis Using an ANN

To predict death status for cirrhosis, an ANN was created through Multi-Layer Perceptron with a hyperbolic tangent as a hidden layer activation function and softmax as an outer layer activation function, as well as cross-entropy as the error function. The standardized method was used for the rescaling of continuous variables, and 72.4% (226 datapoints) of the dataset was used for training and the remaining 27.6% (86 datapoints) was used for testing purposes. The number of hidden layers was one, the number of units in the hidden layer was four (excluding the bias unit), and the activation function for the hidden layer was a hyperbolic tangent. For the output layer, the number of units was two, the activation function was softmax, and the error function was cross-entropy. IBM SPSS software version 29 was used to analyze the data using the ANN method. 

**Figure 3:**
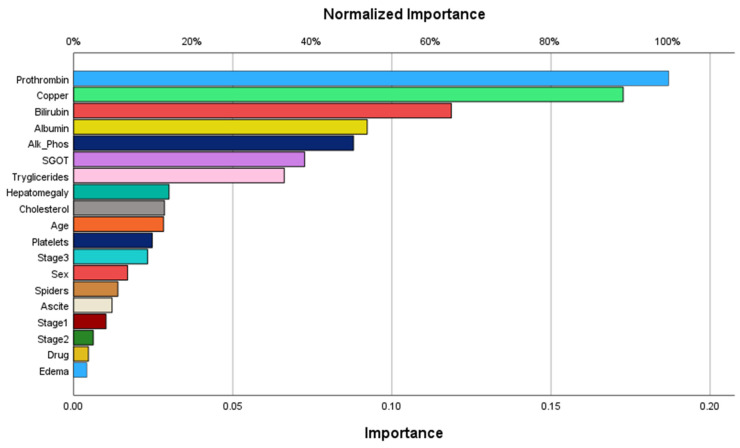
Normalized importance of predictor variables.

The order of importance of predictor variables according to the ANN is shown in Figure 3, and the importance and normalized importance values are given in Table 3. 

The most important variable identified by the ANN was prothrombin, followed by copper, bilirubin, albumin, alkaline phosphatase, and SGGT. The least important variable was edema. 

**Figure 4:**
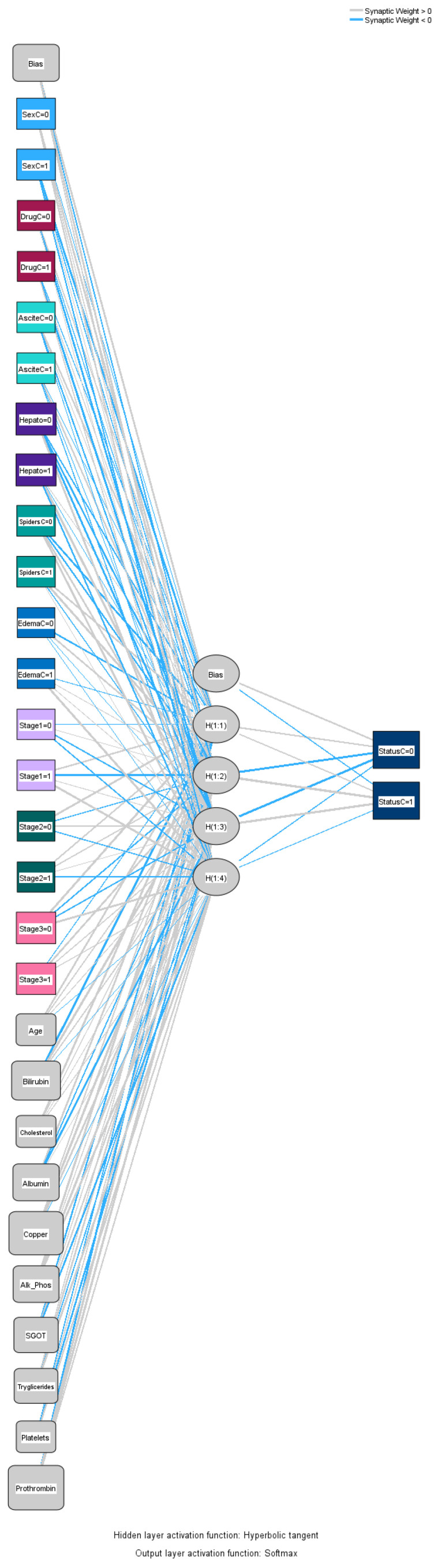
Graphical representation of the ANN.

**Figure 5:**
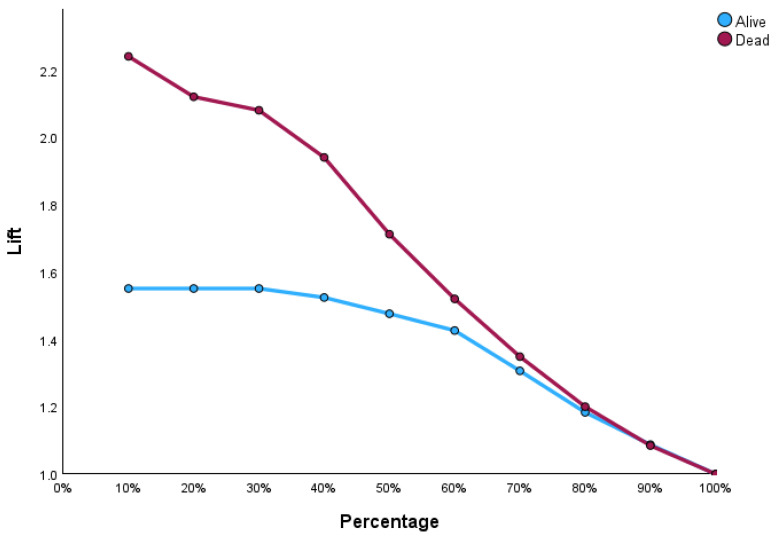
Lift graph for the ANN.

Figure 4 illustrates the input–output relations with hidden multilayers, which performs all the calculations to find hidden features and patterns. To assess the performance of the ANN, we will use lift and gain diagrams. A lift diagram displays the predictive ability of a binary ANN classification model. It measures how effectively the ANN identifies positive instances compared to a baseline of random selection. Furthermore, it indicates how much better one can expect to do with the predictive model compared to without a model. Figure 5 shows the lift curve. For the first 10% of the data, the lift is approximately 2.25, meaning that selecting 10% of the observations with the highest predicted values using the ANN model results in 2.25 more deaths than if that 10% were selected at random. 

**Figure 6:**
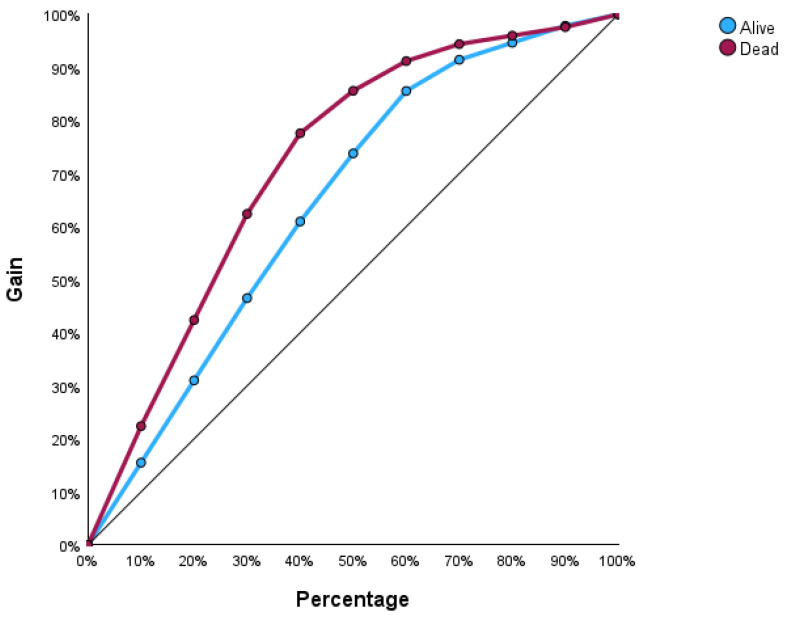
Gain curve for the ANN.

The gain at a given decile level is the ratio of cumulative number of deaths up to that decile to the total number of deaths in the entire dataset. For instance, we can identify and target approximately 23% of patients who died as a result of cirrhosis by sampling only 10% of those patients, as shown in Figure 6.

It was found that in the testing stage the accuracy of the ANN was 80.2%. The true positive rate (TPR), which is also known as recall, was 74.2%. The precision (PRE) of the ANN was 71.9%, with an F-score (FS) of 73.0%. The area under this curve (AUC) was found to be 0.871 for both the training and testing sets. The AUC sums up how well a model can produce relative scores to discriminate between positive or negative instances across all classification thresholds. The AUC ranges from 0 to 1, where 1 indicates a perfect model and 0.5 shows random guessing. The calculated metrics for each ML classification method are shown in Table 4.

#### 3.1.2. Analysis of Cirrhosis Using LR

For the LR, the model accuracy was 82.1%. The TPR was 72.8%. The precision was 80.5% with an FS of 76.5%. The AUC was found to be 0.898. The value of -2log-likelihood for LR was 255.884 and the Akaike Information Criterion (AIC), which is a measure of model quality, was 295.884. The Bayesian Information Criterion (BIC), which is a criterion for model quality, was 370.7440638. Significant variables were prothrombin (*p*-value < 0.001), age (*p*-value = 0.002), alkaline phosphatase (*p*-value = 0.004), and SGOT (*p*-value = 0.037). 

#### 3.1.3. Analysis of Cirrhosis Using PA

The accuracy of the PA model was 81.7%, with a TPR value of 70.4%. The precision was 81.5% with an FS of 75.5%. The ROC curve for PA has an AUC value of 0.896. The value of -2log-likelihood for LR was 260.274 and the AIC was 300.273. The BIC was 375.134. Significant variables were prothrombin (*p*-value < 0.001), age (*p*-value = 0.003), alkaline phosphatase (*p*-value = 0.003), and SGOT (*p*-value = 0.045). Significant variables were prothrombin (*p*-value < 0.001), age (*p*-value = 0.003), alkaline phosphatase (*p*-value = 0.003), and SGOT (*p*-value = 0.045).

#### 3.1.4. Analysis of Cirrhosis Using Random Forest

To perform the random forest analysis for cirrhosis data, the R package randomForest was used. Breiman’s random forest algorithm was used for classification [21,22]. The accuracy of random forest in classifying cirrhosis death was 79.2%. The recall was 72.0% and the precision was 75.0%. The FS for the random forest was 73.5%, with an AUC value of 0.852. 

**Figure 7:**
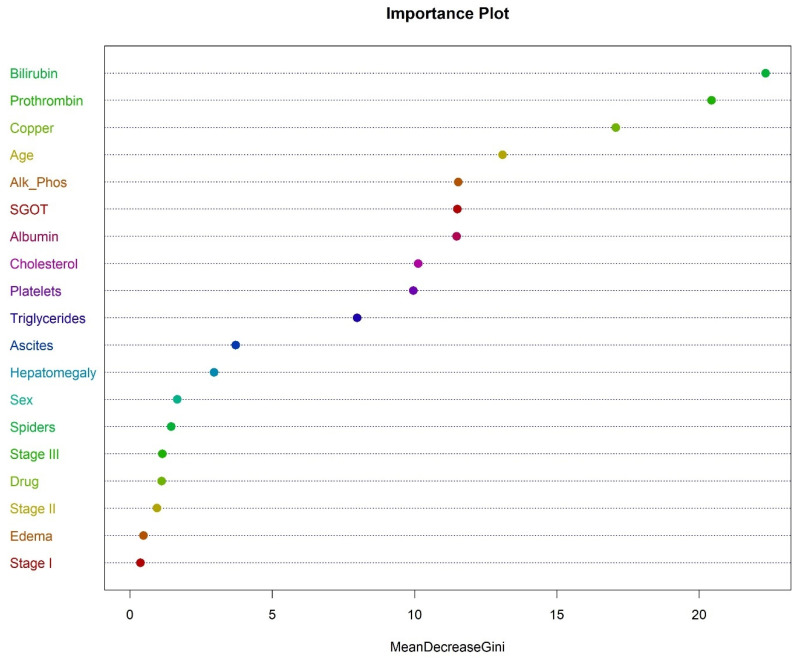
Importance plot for random forest.

The most important variable in classifying the death due to cirrhosis was bilirubin, followed by prothrombin, copper, age, alkaline phosphatase, and SGOT, as shown in Figure 7. 

**Figure 8:**
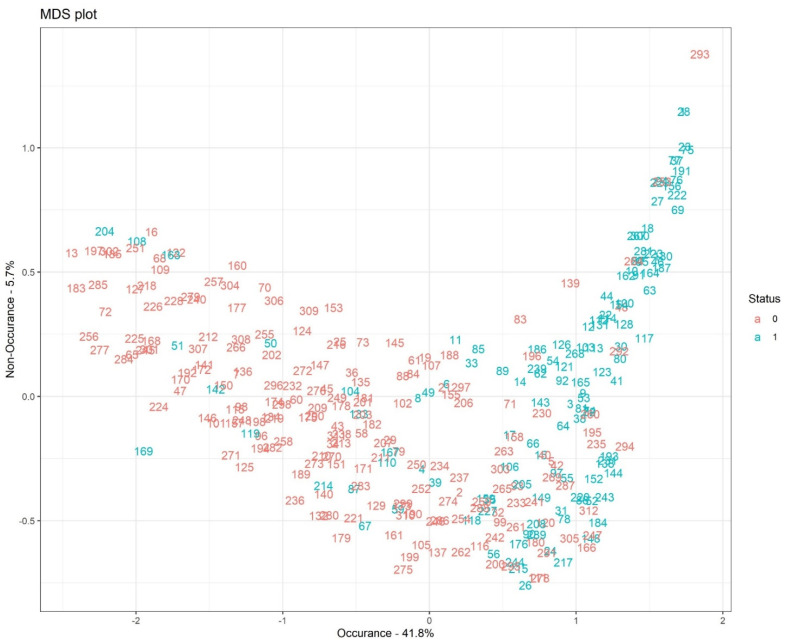
MDS plot.

Figure 8 illustrates the multidimensional scaling (MDS) plot based on proximity measures across observations with 41.8% occurrence and 5.7% non-occurrence rates. The MDS plot shows a pictorial visualization of the level of similarity of individual cases, coded here as 1 (dead) and 0 (alive) in our dataset.

**Figure 9:**
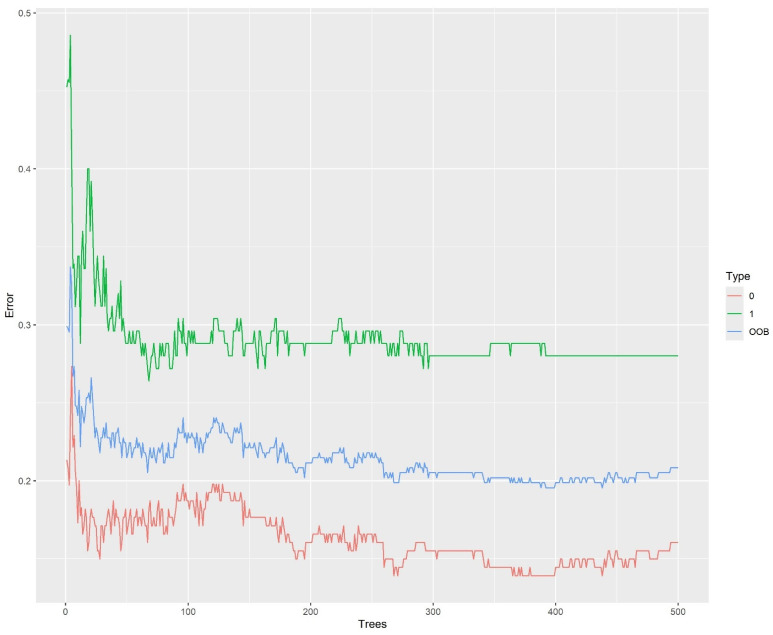
Error plot.

Figure 9 shows the error plot, which gives a visualization of the “Out-of-Bag (OOB) error”. It is a way to estimate random forest’s prediction error by using datapoints that were not used in the training stage for each individual tree within the forest. 

#### 3.1.5. Analysis of Cirrhosis Using Taba Regression

**Figure 10:**
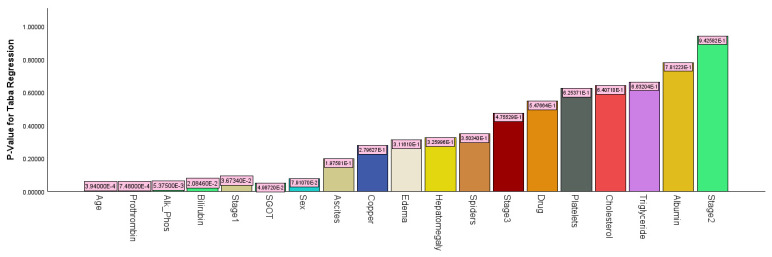
Bart chart for *p*-values using binary Taba regression.

An analysis of cirrhosis data using Taba regression resulted in a recall of 76.0% and a precision of 79.2%. The accuracy was 82.4%, with an FS of 0.775 and an AUC value of 0.902. The -2log-likelihood for binary Taba regression was 244.884, with an AIC value of 284.584 and a BIC value of 359.444. As shown in Figure 10, the most significant predictor variable was age (*p*-value < 0.001), followed by prothrombin (*p*-value < 0.001), alkaline phosphatase (*p*-value = 0.021), stage 1 (*p*-value = 0.037), and SGOT (*p*-value =0.050). Table 5 gives the parameter estimates for binary Taba regression. 

## 4. Discussion

The performance of any given classification method differs wildly depending on the nature of the dataset. Selecting a model to implement for a particular application on the basis of performance measures still remains a major topic of discussion in classification. In the cirrhosis dataset, the standard measures of classification considered in this paper, such as recall, precision, F-score, and AUC, showed that Taba performs extremely well in comparison to four other popular ML classification methods. These measures offer intuition into the model performance as well as help in finding the most appropriate model for the analysis of the dataset under consideration. Taba regression performed better, with higher overall accuracy, TPR, FS, and AUC value compared to the other methods considered in this study.

ANNs and RF necessitate more computational tools and data to perform well. For instance, RF builds multiple decision trees and combines their results, which requires significant computational resources. This collective approach makes it slower than Taba regression. Training an RF model can take a long time, which may not be ideal for real-world applications. This computational intensity can also lead to high memory usage and stress systems with limited resources. 

Taba regression uses less processing power and memory, has computational efficiency in that it is faster to train, has fewer parameters to tune, is less prone to overfitting, and has the flexibility to handle binary, multinomial, and ordinal categorical outcome variables. These characteristics allow Taba regression to work well with smaller datasets or when the data have high variance, noise, or influential observations in the explanatory variables. Unlike ANNs and RF, Taba regression outputs probabilities, which helps in understanding the relationship between the outcome and explanatory variable(s) and the confidence level of predictions. Taba regression is easy to understand, implement, and interpret, making it a desirable method when analyzing data.

## 5. Conclusions

A new classification method for the analysis of binary, multinomial, and ordinal outcome was introduced. Taba regression performed best with regard to accuracy, recall, FS, and AUC when compared with the other four models considered in this paper. The results indicate that Taba regression is a competitive ML method when compared with ANNs, RF, LR, and PA in classifying death status using liver cirrhosis data. Based on the performance of Taba regression, we hope researchers will consider using this model as a classification model when analyzing their data. In the future, we plan to (1) develop an R program capable of performing all necessary computations for binary, multinomial, and ordinal Taba regressions, (2) evaluate the performance of multinomial and ordinal Taba regressions in comparison with other ML methods using biomedical or public health data, and (3) evaluate the robustness of these ML techniques under deterministic and random noise for small, medium, and large datasets. Our data reveal that Taba achieves high accuracy with respect to ML metrics and offers a practical and accessible solution for tackling classification problems, effectively balancing interpretability and accuracy in classification tasks.

## Figures and Tables

**Table 1:** Frequency statistics of categorical variables.

Variable		N	Percent
Death Event	Alive	187	59.9%
	Dead	125	40.1%
Drug	D-Penicillamine	158	50.6%
	Placebo	154	49.4%
Ascites	No	288	92.3%
	Yes	24	7.7%
Hepatomegaly	No	152	48.7%
	Yes	160	51.3%
Spiders	No	222	71.2%
	Yes	90	28.8%
Sex	Female	276	88.5%
	Male	36	11.5%
Edema	N or Y	283	90.7%
	S	29	9.3%
Stage	I	16	5.1%
	II	67	21.5%
	III	120	38.5%
	IV	109	34.9%

**Table 2:** Descriptive statistics of continuous variables.

Variable	N	Minimum	Maximum	Mean	S.D.
Age (in days)	312	9598	28,650	18,269.44	3864.805
Bilirubin	312	0.3	28.0	3.256	4.530
Cholesterol	312	−130	1775	368.75	233.063
Albumin	312	1.96	4.64	3.520	0.41989
Copper	312	−93	588	97.04	86.019
Alkaline Phosphatase	312	289	13,862	1982.66	2140.389
SGOT	312	26	457	122.56	56.700
Triglycerides	312	6	598	124.88	65.713
Platelets	312	62	563	262.39	95.551
Prothrombin	312	9.0	17.1	10.726	1.004

**Table 3:** Predictor variable normalized importance.

Variable	Importance	Normalized Importance
Sex	0.017	9.1%
Drug	0.005	2.5%
Ascites	0.012	6.5%
Hepatomegaly	0.030	16.0%
Spiders	0.014	7.4%
Edema	0.004	2.2%
Stage I	0.010	5.4%
Stage II	0.006	3.3%
Stage III	0.023	12.4%
Age	0.028	15.1%
Bilirubin	0.119	63.5%
Cholesterol	0.029	15.3%
Albumin	0.092	49.3%
Copper	0.173	92.4%
Alkaline phosphatase	0.088	47.0%
SGOT	0.073	38.8%
Triglycerides	0.066	35.4%
Platelets	0.025	13.2%
Prothrombin	0.187	100.0%

**Table 4:** Metrics of the Taba, LR, PA, ANN, and RF models.

	TPR	TNR	FPR	FNR	PRE	FS	Accuracy	AUC
Taba	0.760	0.870	0.130	0.240	0.792	0.775	0.824	0.902
LR	0.728	0.882	0.176	0.182	0.805	0.765	0.821	0.898
PA	0.704	0.893	0.107	0.296	0.815	0.755	0.817	0.896
ANN	0.742	0.836	0.164	0.258	0.719	0.730	0.802	0.871
RF	0.720	0.840	0.160	0.280	0.750	0.735	0.792	0.852

**Table 5:** Parameter estimates for binary Taba regression.

Parameter		Estimate	SE	t Value	*p*-Value
Constant		−5.661999	1.417977	−3.993013	0.000081
Age		0.000075	0.000021	3.582989	0.000394
Sex	Female	−0.438779	0.249072	−1.761652	0.079107
	Male	ref			
Drug	D-Penicillamine	0.079277	0.131706	0.601920	0.547664
	Placebo	ref			
Ascites	Yes	2.102162	1.628034	1.291227	0.197581
	No	ref			
Hepatomegaly	Yes	0.150874	0.153365	0.983760	0.325996
	No	ref			
Spiders	Yes	0.150437	0.160839	0.935328	0.350343
	No	ref			
Edema	S	−0.262751	0.259253	−1.013494	0.311610
	N or Y	ref			
Stage1	Y	−1.267696	0.604319	−2.097725	0.036734
	N	ref			
Stage2	Y	−0.014499	0.201138	−0.072082	0.942582
	N	ref			
Stage3	Y	0.122902	0.172041	0.714376	0.475529
	N	ref			
Bilirubin		0.103430	0.044533	2.322553	0.020846
Cholesterol		0.000212	0.000455	0.467162	0.640710
Albumin		0.055789	0.200705	0.277965	0.781223
Copper		0.001260	0.001164	1.083040	0.279627
Alkaline Phosphatase		0.000110	0.000039	2.803323	0.005375
SGOT		0.002870	0.001458	1.968708	0.049872
Triglyceride		−0.000577	0.001325	−0.435909	0.663204
Platelets		−0.000392	0.000802	−0.488737	0.625371
Prothrombin		0.316535	0.092958	3.405133	0.000748

## Data Availability

The data presented in this study are available in UC Irvine Machine Learning Repository at doi.org/10.24432/C5R02G (accessed on 22 March 2023). These data were derived from the following resources available in the public domain: https://archive.ics.uci.edu/dataset/878/cirrhosis+patient+survival+prediction+dataset-1 (accessed on 22 March 2023).
